# Excessive MALAT1 promotes the immunologic process of neuromyelitis optica spectrum disorder by upregulating BAFF expression

**DOI:** 10.1515/tnsci-2022-0306

**Published:** 2023-10-17

**Authors:** Jing Chen, Lijie Zhang, Jingyu Lin, Zeng Wang, Aiyu Lin

**Affiliations:** Department of Neurology and Institute of Neurology of the First Affiliated Hospital, Institute of Neuroscience, and Fujian Key Laboratory of Molecular Neurology, Fujian Medical University, Fuzhou 350005, China; Department of Neurology, National Regional Medical Center, Binhai Campus of the First Affiliated Hospital, Fujian Medical University, Fuzhou 350212, Fujian, China; Department of Neurology, Zhangzhou Affiliated Hospital of Fujian Medical University, Zhangzhou, 363000, Fujian, China; Department of Neurology, National Regional Medical Center, Binhai Campus of the First Affiliated Hospital, Fujian Medical University, Fuzhou 350212, China; Central Research Lab, The First Affiliated Hospital, Fujian Medical University, Fuzhou, 350005, Fujian, China

**Keywords:** neuromyelitis optica spectrum disorder, metastase-associated lung adenocarcinoma transcription 1, miR-30b-5p, mononuclear macrophages, B cell activating factor

## Abstract

Increased B cell activating factor (BAFF) expression in patients with neuromyelitis optica spectrum disorder (NMOSD) is associated with B cell overstimulation, but the underlying mechanism remains unclear. This study aimed to reveal the emerging mechanisms that regulate BAFF expression in the inflammatory process of NMOSD. The results showed that the expression of miR-30b-5p was significantly decreased in NMOSD CD14^+^ monocytes compared with the normal control. Furthermore, we confirmed that metastasis-associated lung adenocarcinoma transcription 1 (MALAT1) is an upstream target of miR-30b-5p, and it could act as a ceRNA and absorb miR-30b-5p with reduced expression of miR-30b-5p. The low expression of miR-30b-5p could not bind to BAFF messenger RNA (mRNA), which resulted in the overexpression of both BAFF mRNA and protein expression. Overexpression of BAFF could bind to the corresponding receptors on B cells, which may initiate activation and proliferation of B cells and increase their production of autoantibodies. Therefore, these findings interpreted that excessive MALAT1 expression in NMOSD mononuclear macrophages led to increased BAFF expression by targeting miR-30b-5p, which caused B cell autoimmune reaction and autoantibodies production, aggravated the disease progression of NMOSD.

## Graphical abstract

**Figure:**
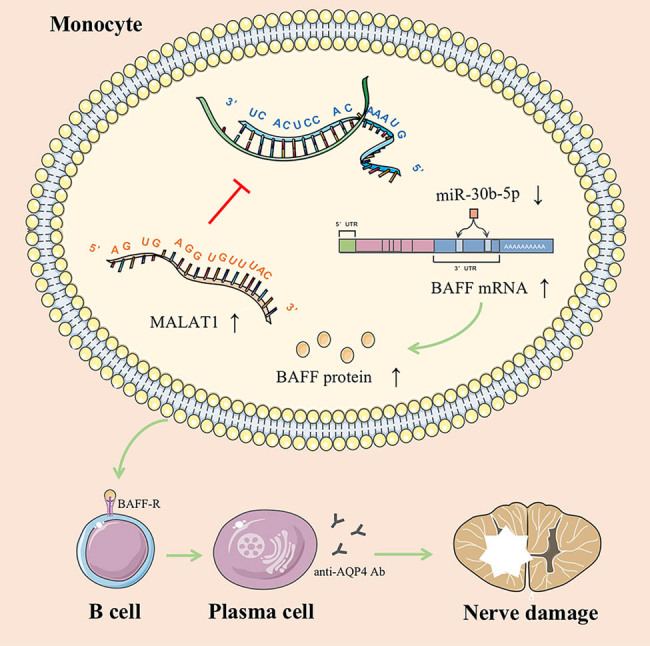

## 1 Introduction

Neuromyelitis optica spectrum disorder (NMOSD) is a demyelinating disease of the central nervous system characterized by severe involvement of the optic nerve and the spinal cord, frequent recurrence, and high disability, which tends to onset in middle-aged women [1]. The current evidence suggested that humoral immune abnormalities play an important role in the pathogenesis of NMOSD. Aquaporin4 antibody (AQP4-Ab) has been proved to be specific for NMOSD [2]. In addition, cytokine imbalance is common in patients, and an increase in the concentration of pro-inflammatory cytokines has been reported in the CSF and serum of patients with NMOSD [3–5]. However, the mechanism of signal crosstalk between cytokines and immune cells in NMOSD is still unknown, which is essential for intervening in the corresponding immune inflammatory damage.

Protein-coding genes have been the focus of research in the past decades, while non-coding RNA have been considered as “garbage” and “noise” generated in the transcription process, but there is increasing evidence that these so-called “by-products” are also involved in cell and tissue growth and homeostasis [6]. MicroRNA has been proven to have a strong regulatory role in many inflammatory reactions or autoimmune diseases [7]. It plays an irreplaceable role in tumors and immunity, and is expected to become diagnostic markers and therapeutic targets of diseases. In the previous study, it was found that miR-30b-5p in the whole blood of NMOSD patients was significantly down-regulated which was statistically significant compared with multiple sclerosis (MS) and the normal control group [8], and was consistent with the results of Keller et al. [9]. Nevertheless, little is known about the role of miR-30b-5p in the biological function of immunocyte and the pathogenesis of autoimmune diseases.

Metastase-associated lung adenocarcinoma transcription 1 (MALAT1), also known as NEAT2, is an 8 KNT long non-coding RNA (IncRNA) located on 11q13 and was originally considered as a poor prognostic parameter for lung cancer patients [10,11]. In recent years, MALAT1 has been found to affect the innate immune response process by affecting the polarization of mononuclear macrophages [12,13]. Meanwhile, emerging evidence supported increase expression of MALAT1 mainly enriched in inflammatory cytokine pathways [14,15]. Therefore, attempts to understand the molecular mechanism of MALAT1 in the mononuclear macrophage cell line of NMOSD is needed for finding its potential pathogenesis.

BAFF is a key survival factor of peripheral blood B cells, and overexpression of BAFF can be found in various autoimmune diseases [16,17]. BAFF secreted by monocytes has been reported to promote pathogenic AQP4 antibody production, which is essential for the development and progression of NMOSD [18]. BAFF is significantly elevated in serum and cerebrospinal fluid of NMOSD patients, and upregulating BAFF is thought to promote and maintain the production of AQP4-Ab [19,20], thereby preventing upstream signaling, then reducing the production of BAFF, which may benefit from the disease progression of NMOSD.

Herein, this study aims to elucidate whether MALAT1/miR-30b-5p/BAFF signal axis is involved in the central nerve injury of NMOSD following immune disorder as well as the underlying mechanisms.

## 2 Materials and methods

### 2.1 Participants

Between September 2017 and September 2021, a total of 38 patients were enrolled in this study, all from the First Affiliated Hospital of Fujian Medical University, following 2015 NMOSD diagnostic criteria developed by the international NMO Diagnostic Group (IPND) [21], including 35 patients who were AQP4-IgG positive and three patients were MOG-IgG positive (CBA assay, in house). Clinical data such as duration of disease, frequency of attack, treatment plan, and EDSS score were presented in Table 1. All patients were in the acute stage. Thirty-eight healthy control groups were matched by sex and age. According to recent studies, MOG antibody-associated disease (MOG-AD) has been separated from NMOSD disease as a new disease entity [22–24]; therefore, the NMOSD patients referred to in this research only include AQP4 antibody-positive patients. Total transcriptome sequencing was performed in three pairs of untreated NMOSD patients and matched healthy controls. All of the above patients were from the clinical cohort (NCT: 04386018).

**Table 1:** Clinical and laboratory characteristics of participants in the study

Characteristics	Patients (*n* = 38)	Control (*n* = 38)
Age, mean ± SD year	38.7 ± 11.0	37.4 ± 10.0
Female/male ratio	32/6	35/3
Time since first event (days)	1987.4 ± 1362.3	—
Recurrence rate (＜3/≥3)	26/12	—
Disease localization		
Optic neuritis	6/38	—
Myelitis	26/38	—
Brain	14/38	—
Treatment		
Corticosteroids	33/38	—
Gamma globulin	2/38	—
Azathioprine	2/38	—
EDSS score (median[range])	3 (1–5)	—
Ratio of AQP4-IgG positivity (±)	35/38	—
Ratio of MOG-IgG positivity (±)	3/38	—
Ratio of autoantibody positivity (±)	8/38	—

### 2.2 CD14^+^ cells isolation

From NMOSD patients and controls, 10 mL of blood was collected in vacuette containing ethylenediaminetetraacetic acid and immediately processed according to biosafety rules. Peripheral blood mononuclear cells (PBMCs) were isolated on a Ficoll density gradient (TBDscience, China). All PBMC samples were studied fresh (operative time ≤6 h), which were classified into CD14^+^ cells bead by positive magnetic separation (Miltenyi Biotec, Germany) according to the manufacturer’s instructions. The sorted cells were determined to have purity greater than 90% by flow cytometry.

### 2.3 Whole transcriptome sequencing

Three untreated NMOSD patients in the acute stage of the disease and three age–gender matched healthy controls were included. After extracting the total RNA, double-stranded cDNA was synthesized and purified. After terminal repair, a ring connector was connected at both ends of the double-stranded cDNA under the action of DNA ligase, and then the ring was opened by USER enzyme and the second strand of cDNA was disconnected. AMPure XP Beads (Beckman Coulter, Inc.) are used to screen the fragment length to make the target fragment length in the range of 300–400 bp. High-fidelity DNA polymerase is used for PCR amplification and AMPure XP Beads were purified. After quality inspection, the purified library was sequenced by Illumina HiSeq sequencing platform. Clean data were obtained by filtering the sequencing data and compared with the specified reference genome for analysis of transcription/gene expression and differential expression of transcription/gene. The databases involved include Ensemble, GO, KEGG, and STRING. Application software included FastQC, Cutadapt, HISat2, SAMtools, Stringtie, DESeq2, BCFTools, MISA, rMATS, CIRCexplorer, CPAT, R, and Cytoscape.

### 2.4 Dual-luciferase assay

The promoter validity and miRNA target genes were verified by luciferase double reporter assay. Since miRNA mainly acts on the 3′-UTR of the target gene, the MALAT1 3′-UTR region was constructed behind the reporter gene Luciferase in the vector (Table S1). After comparing the overexpression or interference of miR-30b-5p, changed in reporter gene expression (monitoring changes in luciferase activity) could quantitatively reflect the interaction ability between miR-30b-5p and MALAT1. Combined with site-directed mutation, their action sites were further identified. Plasmid construction: vector: GV272 (Jikai), XbaI/XbaI digestion (Jikai).

HEK293T cells were cultured to logarithmic growth stage, made cell suspension, counted, inoculated in a 24-well culture plate (about 105 cells), and cultured in 5% CO_2_ incubator at 37°C until the cell fusion degree reached about 60%. The constructed plasmid was transfected with X Tremegene HP (ROCHE) transfection reagent. The expression of fluorescent-labeled genes on the plasmid was observed 24–48 h after transfection to determine the transfection efficiency. Luciferase (Dual-Luciferase^®^ Reporter Assay System, Promega) was detected 48 h after transfection. Firefly luminescence and Renilla luminescence were measured with a microplate tester.

The experimental data in this study were presented as the mean ± standard deviation (SD) of three independent experiments. The statistical significance of the differences in the experimental data was valued by ANOVA followed by Tukey’s *post hoc* test. The statistical significance level was set as *P ＜ 0.05, **P ＜ 0.01, and ***P ＜ 0.001.

### 2.5 Quantitative PCR of participant whole blood cells and monocytes

To further confirm the expression levels of miR-30b-5p, MALAT1, and BAFF, total RNA including non-coding RNAs was extracted from the whole blood cells and sorted CD14^+^ cell of NMOSD patients and matched with the healthy controls by miRNeasy extraction kit (Qiagen, Valenica, CA) using QIAzollysis reagent according to the manufacturer’s instructions. Concentration of RNA was determined using NanoDrop2000 which is very accurate to measure even the small quantities of RNA (Thermo Scientific, USA). Reverse transcription was carried out on extracted RNA in a final volume 20 μL reactions using RT2 first strand kit (Qiagen, Valenica, CA) according to the manufacturer’s instructions. Quantitative PCR was performed with the TaqMan Gene Expression Master Mix (Applied Biosystems) using primers against miR-30b-5p, MALAT1, and BAFF (Table S2). mRNA levels were standardized to the reference human β-actin and GAPDH Endogenous Control (Applied Biosystems). Real-time PCR was done on 20 μL reaction mixture using Rotor geneQ System (Qiagen) with the following conditions: 95°C for 10 min, followed by 45 cycles at 95°C for 15 s and 60°C for 60 s. Gene expression relative to internal control (2^−ΔΔCt^) was calculated. Fold change was calculated using 2^−ΔΔCt^ for relative quantification. At the same time, we also analyzed the correlation between the three.

### 2.6 Construction of miR-30b-5p cell lines overexpression and knockdown

THP-1 cells were cultured in medium containing 10% fetal bovine serum at 37°C under 5% CO_2_, and transfected with plasmid during logarithmic growth phase. THP-1 cells were divided into miR-30b-5p Nor group (transfected with empty vector), miR-30b-5p KD group (transfected with LV-has-mir-30b-5p-inhibition[43448-1]), and miR-30b-5p OE group (transfected with LV-has-mir-30b-5p[41827-3]). The cells were inoculated at a density of 5 × 10^5^/well and cultured in a cell incubator containing 5% CO_2_ at 37°C. Lipofectamine 2000 (Invitrogen, Carlsbad, CA) was used to transfect cells (4 Ig per well) and the cell adhesion integration rate was 80%. Cells were collected 24 h after transfection, and the expression of miR-30b-5p was detected to determine whether the overexpressed and knockdown miR-30b-5p cell lines were successfully constructed. Quantitative PCR method of miR-30b-5p, MALAT1, and BAFF was the same as the method mentioned above. The difference had statistical significance (*P* < 0.01).

### 2.7 Western blot analysis

The above THP-1 cells were lysed in protein lysis buffer containing a proteinase inhibitor (Thermo Fisher Scientific). Lysates were then centrifuged for 15 min at 14,000*g* at 4°C, and the protein concentration was determined using the Bradford Protein Assay (Thermo Fisher Scientific). Proteins were separated by sodium dodecyl sulfate–polyacrylamide gel electrophoresis using 10% polyacrylamide gels and then transferred onto polyvinylidene difluoride membranes (Millipore, MA, USA). The membranes were blocked with 5% non-fat dry milk in Tris-buffered saline containing 0.1% Tween-20 buffer and immunoblotted with primary antibodies, including anti-BAFF (Abcam) and anti-β-actin (Cell Signaling, BSN, USA). The band intensity was then quantified using Quantity One software (Bio-Rad, CA, USA).

### 2.8 Statistical analysis

The data were expressed as mean ± SD. Means of two continuous normally distributed variables were compared by independent samples Student’s *t*-test. Mann–Whitney *U*-test was used, respectively, to compare means of two and three or more groups of variables not normally distributed. Correlations were analyzed using Pearson’s correlation coefficient. Significance was set as *P* < 0.05. All analyses were performed using the SPSS 26.0 software and GraphPad Prism 9. A probability (*P*) value of <0.05 was considered to be significant.


**Ethical approval:** The research related to human use has been complied with all the relevant national regulations, institutional policies and in accordance with the tenets of the Helsinki Declaration, and has been approved by the authors’ institutional review board or equivalent committee. This study was approved by the ethics commission of the first affiliated hospital of Fujian Medical University.
**Informed consent:** Informed consent has been obtained from all individuals included in this study.

## 3 Results

### 3.1 MALAT1 was aberrantly upregulated expression in patients with NMOSD

**Figure 1:**
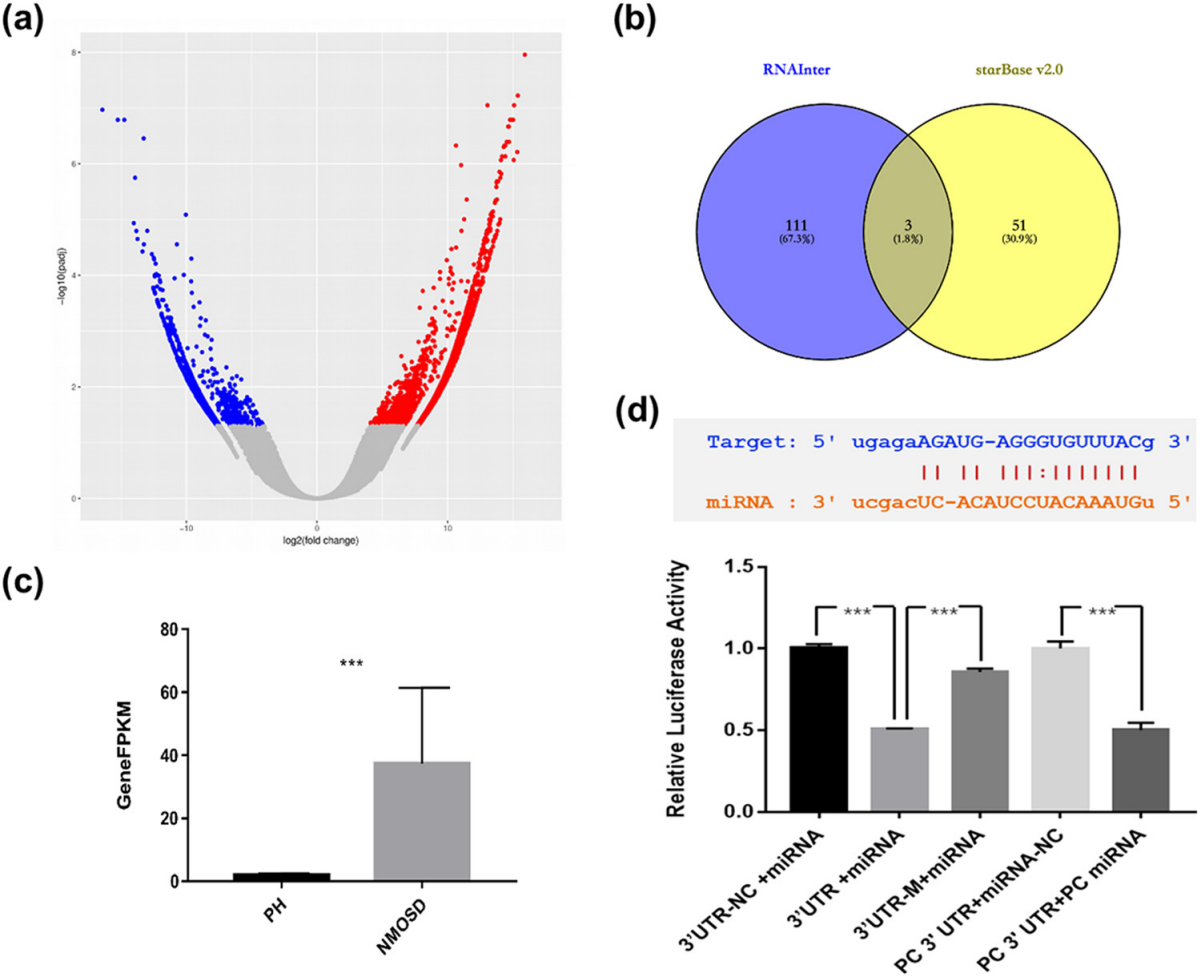
MALAT1 was overexpressed in NMOSD patients and had binding site with miR-30b-5p. (a) 247574 IncRNAs were differentially expressed in the volcano plot of differentially expressed gene, including MALAT1 (top 10). (b) RNAInter and Starbase v2.0 databases were used to predict the target IncRNA of miR-30b-5p, and MALAT1 was one of them. (c) Columnar statistics of FPKM values of MALAT1 in NMOSD and healthy controls (***P < 0.001). (d) Starbase2.0 database showed that MALAT1 (uplink) had a binding site with miR-30b-5p (downlink). The results of the double luciferase reporter gene experiment showed that miR-30b-5p could bind to the 3′-UTR region of MALAT1 and lost its binding ability after site-directed mutation. In positive control, TRAF6 gene 3′-UTR plasmid was used to overexpress hSA-miR-146b vector plasmid (***P < 0.001).

In order to search for the upstream regulatory sites of miR-30b-5p, we performed whole transcriptome sequencing based blood in three pairs of patients with NMOSD and matched healthy control. Using the criteria of >2-fold change, *P* < 0.05, and the threshold of fragments per kilobase of transcript per million (FPKM) mapped reads >50, we identified a total of 247,574 IncRNAs that were differentially expressed between patients and healthy control (Figure 1(a)). The results showed that the expression level of MALAT1 in NMOSD patients was significantly higher than that in healthy controls after difference analysis on IncRNA level (Figure 1(c)).

### 3.2 MALAT1 was interacted with miR-30b-5p

MALAT1 screened by whole transcriptome sequencing and miR-30b-5p noted in previous studies were predicted in starbase2.0 database and the RNAInter database, and both were found to have binding sites (Figure 1(b)). At the same time, Luciferase reporter gene assay was carried out. The sequence of MALAT1 3′-UTR region was constructed into Luciferase gene of GV272 vector, and then transfected into cells. After overexpression or interference of miR-30b-5p, the expression of Luciferase reporter gene was detected. The interaction between miR-30b-5p and MALAT1 could be quantitatively reflected by using sea kidney luciferase as an internal reference gene. Meanwhile, the sites of action were further identified by site-directed mutation. Experimental results confirmed that MALAT1 could bind to miR-30b-5p and had an interaction relationship (Figure 1(d)).

### 3.3 Increased accumulation of MALAT1 upregulated BAFF expression by the signaling pathway of miR-30b-5p in the whole blood cells and CD14^+^ monocytes of NMOSD patients

**Figure 2:**
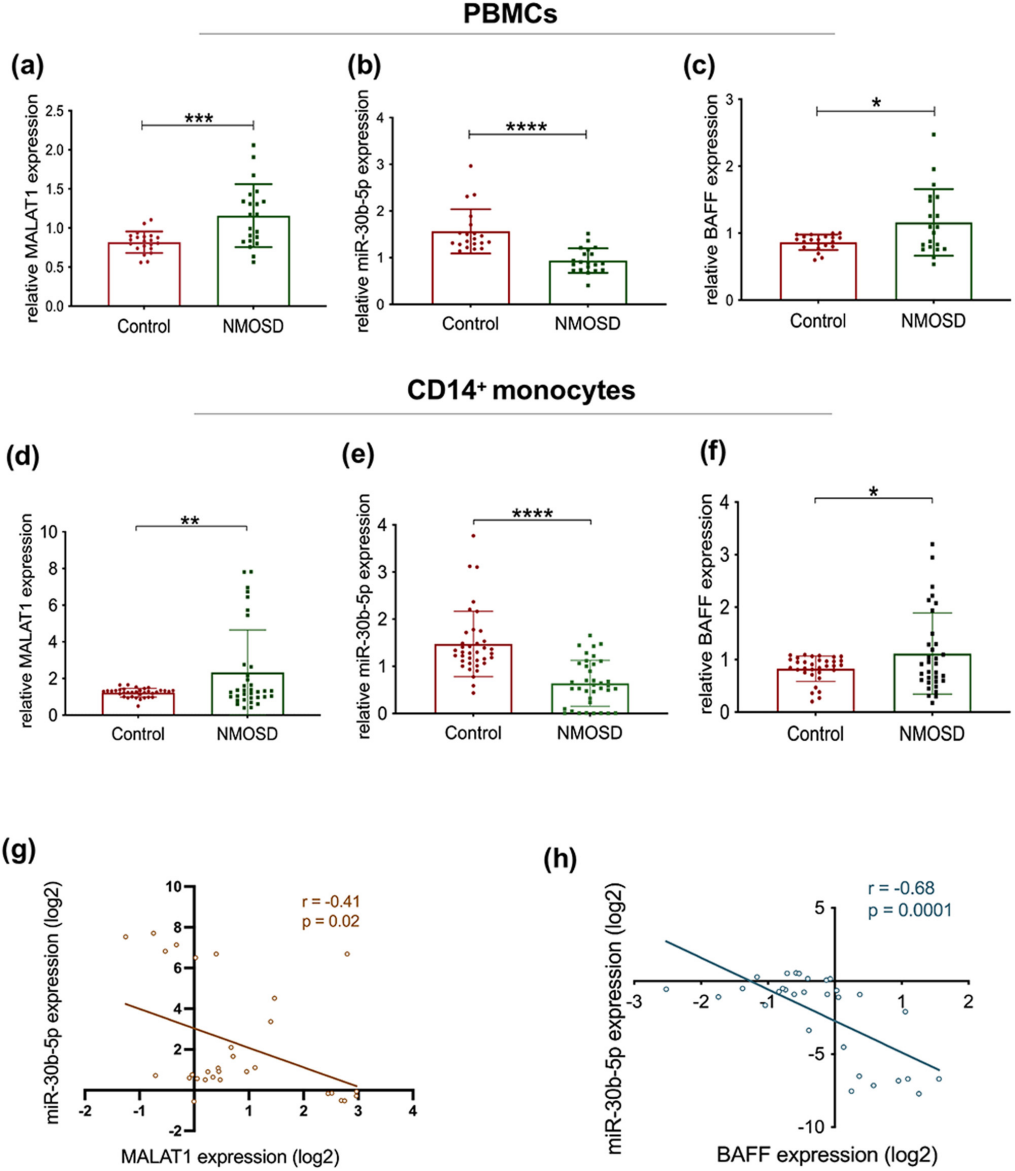
Expression and relationship of MALAT1, miR-30b-5p, and BAFF in the whole blood cells and CD14^+^ monocytes of NMOSD patients in qPCR assay. (a–c) 2^−△△CT^ values of MALAT1, miR-30b-5p, and BAFF in the whole blood cells in control group and NMOSD group, respectively (*P < 0.05, ***P < 0.001, ****P < 0.0001). (d–f) 2^−△△CT^ values of MALAT1, miR-30b-5p, and BAFF in CD14^+^ monocytes in control group and NMOSD group, respectively (*P < 0.05, **P < 0.01, ****P < 0.0001). (g) Correlation between the expression of MALAT1 and miR-30b-5p in CD14^+^ monocytes. (h) Correlation between the expression of BAFF and miR-30b-5p in CD14^+^ monocytes.

**Figure 3:**
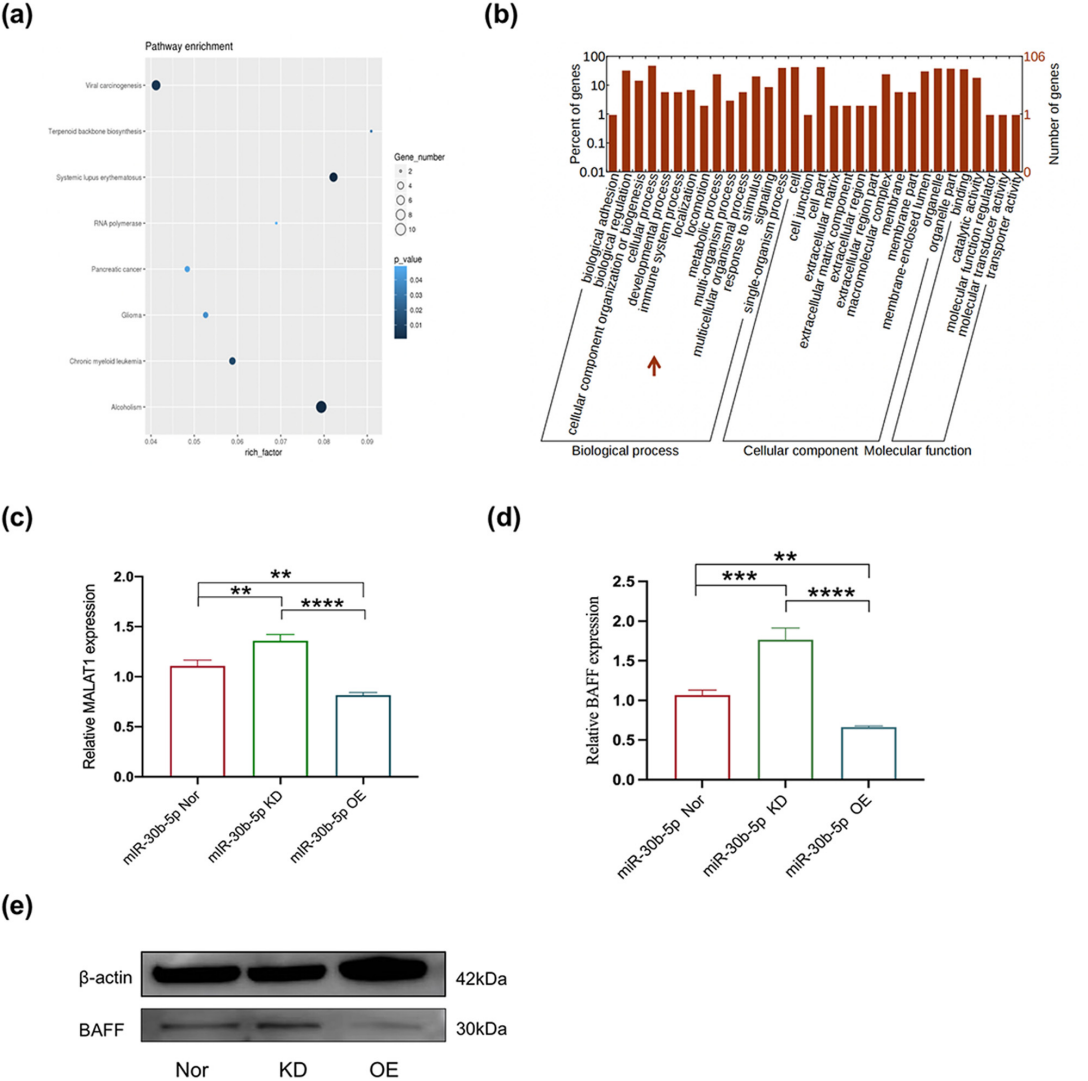
Bioinformatic analysis of whole transcriptome sequencing, then quantitative PCR assay and western blot analysis for BAFF in miR-30b-5p OE group, miR-30b-5p KD group, and miR-30b-5p Nor group. (a) KEGG analysis and (b) GO analysis. (c) The respective bar graphs showed that the expression of MALAT1 gene was increased in miR-30b-5p KD group, and the expression of MALAT1 gene was decreased in miR-30b-5p OE group (**P < 0.01, ****P < 0.0001). (d) The respective bar graphs showed that the expression of BAFF gene was increased in miR-30b-5p KD group, and the expression of BAFF gene was decreased in miR-30b-5p OE group (**P < 0.01, ***P < 0.001, ****P < 0.0001). The cellular status after transfection with LV-has-miR-30b-5p-inhibition[43448-1] and LV-has-miR-30b-5p[41827-3] for 72 h. (e) Representative western blot for BAFF among three group (miR-30b-5p Nor, miR-30b-5p KD, and miR-30b-5p OE).

To further understand the mechanism of action of MALAT1 and miR-30b-5p in the disease process of NMOSD, expression in whole blood cells of 35 NMOSD patients and healthy controls were recruited for preliminary evaluation. Interestingly, MALAT1 and BAFF was overexpressed (Figure 2(a) and (c)), while miR-30b-5p expression was decreased (Figure 2(b)). Combined with functional annotation and signaling pathway prediction by whole transcriptome sequencing, the results showed that the relevant target genes were mainly enriched in pathways similar to systemic lupus erythematosus (SLE) immune inflammation, chronic myeloid cytopathy, and viral infection cancerous lesions (Figure 3(a) and (b)), which was why we focused more on inflammatory alterations in NMOSD myeloid immune cells, and in connection with previous literature reports, CD14^+^ monocytes became the breakthrough point for the next study.

The relationship between MALAT1 and miR-30b-5p was extended to monocyte validation in patients, then we studied whether BAFF was identified as a downstream molecular target of both miR-30b-5p. Real time PCR results showed that MALAT1 and BAFF were significantly increased in CD14^+^ cells of NMOSD patients (Figure 2(d) and (f)), while miR-30b-5p was significantly decreased (Figure 2(e)). Meanwhile, the expression of MALAT1 was negatively correlated with miR-30b-5p (*r* = −0.41, *P* = 0.02, Figure 2(g)), and the expression of BAFF was negatively correlated with miR-30b-5p (*r* = −0.68, *P* = 0.0001, Figure 2(h)). Experimental evidence suggested MALAT1/miR-30b-5p/BAFF signal axis participated in immunity process of NMOSD monocyte. In this experiment, the purity of CD14^+^ cells sorted by flow-verified magnetic beads can reach over 90% (Figure S1).

In addition, comparing NMOSD patients, in CD14^+^ monocytes from three MOG-AD patients, MALAT1 expression was reduced and miR-30b-5p expression was elevated, while BAFF mRNA overexpression was not significant (*P* = 0.7459, Figure S2).

### 3.4 MALAT1 bound to miR-30b-5p to upregulate BAFF expression and trigger immune inflammatory responses

THP-1 was transfected with different plasmids to construct three cell lines: overexpression of miR-30b-5p THP-1 cell group (miR-30b-5p OE), knockdown of miR-30b-5p THP-1 cell group (miR-30b-5p KD), and empty viral THP-1 cells (miR-30b-5p Nor). The correlation between miR-30b-5p and BAFF was further verified in the above three types of cells. At the RNA level, real time PCR detection showed that the expression of MALAT1 was upregulated in THP-1 cells down-regulated miR-30b-5p, while the expression of MALAT1 was down-regulated in THP-1 cells overexpressing miR-30b-5p (Figure 3(c)). In addition, BAFFmRNA expression was up-regulated in THP-1 cells that down-regulated miR-30b-5p and down-regulated in THP-1 cells that overexpressed miR-30b-5p (Figure 3(d)). At the protein level, down-regulation of BAFF protein expression was found in THP-1 cells that overexpressed miR-30b-5p, while the down-regulation of BAFF protein expression was found in THP-1 cells that knocked down miR-30b-5p (Figure 3(e)).

## 4 Discussion

Monocytes have received increasing attention for their role in exacerbating inflammatory storms in autoimmune diseases such as MS, SLE, and rheumatoid arthritis [25–27]. Currently, NMOSD is believed to be mainly due to B cell-mediated autoimmune diseases, but monocytes and other innate immune cells also play an indispensable role [28,29], and there are still gaps in the molecular mechanism of monocytes’ involvement in humoral immunity of NMOSD. In a previous research, nine candidate miRNAs were isolated for real-time PCR from whole blood from 20 healthy controls, 45 NMOSD, and 17 MS patients. Among the mircoRNA, we found that miR-30b-5p was significantly decreased by about 1/2 in NMOSD patients compared with the control group, and was negatively correlated with the degree of intracranial lesions in NMOSD [8], and Keller et al. found similar change [9].

Past studies have found a microarray analysis of mRNA and IncRNA in peripheral mononuclear cells of patients with NMOSD, and found that there were numerous epigenetic differences in patients with NMOSD compared to healthy people, suggesting that these factors would be key to early diagnosis, appropriate treatment, and better prognosis of patients with NMOSD [30]. By targeting mircoRNA, these lncRNAs play an important role in guiding the development of a variety of immune cells and in controlling the dynamic transcription program of immune cell activation markers [31,32]. In order to understand the molecular mechanism of miR-30b-5p in NMOSD disease, we identified the upstream target of MALAT1 through total transcriptomic data and software prediction results. MALAT1 could act as the ceRNA of miR-30b-5p, thereby inhibiting the expression of miR-30b-5p. Most of the previous studies on MALAT1 were mainly on lung cancer and other tumors [33,34]. Our results refreshed the understanding of MALAT1 in the autoimmune process, and could also explain the corresponding molecular basis of MALAT1 in tumor immune tolerance escape [35,36].

There is a genetic susceptibility to autoimmune diseases, a single-cell RNA sequencing study suggested experimental evidence of increased inflammatory cytokine gene expression in immune cells during the early immune response of NMOSD patients [37], and We were more concerned with the risk of immune-inflammatory predisposition brought by differently expressed genetic information, which was reflected not only in the production of pro-inflammatory cytokines, but also in the signal crosstalk between inflammatory cells brought by the cytokines [38]. What were the downstream immunoinflammatory targets of miR-30b-5p? In a study of Sjogren’s syndrome, miR-30b-5p could negatively regulate the expression of BAFF in monocytes [39]. This caught our attention. Therefore, in this study, we hypothesized that NMOSD miR-30b-5p could also negatively regulate BAFF, while down-regulated miR-30b-5p reversed this process, leading to upregulation of BAFF expression. CD14^+^ monocyte levels in a large number of patients also confirmed our idea. In order to further determine the relationship among the axis of MALAT1–miR-30b-5p–BAFF, the THP-1 cell lines of miR-30b-5p overexpression and knockdown were constructed to validate this potential signaling axis, which sheds light on the future of targeted therapy for NMOSD.

As a mimic of NMOSD disease, a similar correspondence of the MALAT1/miR-30b-5p/BAFF signaling axis does not seem to be found in MOG-AD patients, and we hypothesize that this pathway may occur only in NMOSD patients, which promises to be a means of differential diagnosis between the two. Multiple studies have suggested that the NMOSD lesions were more severely involved than those of MOG-AD [40–42], and a recent pathologic research has shown that immune-inflammatory cell infiltration in the acute phase of MOG-IgG experimental autoimmune encephalomyelitis (EAE) mice was less common than in AQP4-IgG EAE, and monocyte macrophage infiltration was not seen, leading to the early onset of inflammation in NMOSD [43], which fitted with our findings that the level of expression of BAFF mRNA, an inflammatory cytokine secreted by monocytes, was slightly lower in MOG-AD patients than in NMOSD patients, and that, because this study enrolled fewer MOG-AD patients, resulting in a slightly weaker level of evidence, and the sample size could be expanded later to clarify the specific regulatory mechanisms involved in the variability.

BAFF, a member of the tumor necrosis factor family that plays an important role in the activation of B cells, was first discovered around 2000 [44,45]. It is mainly divided into two forms: membrane-bound BAFF and soluble BAFF [46,47]. Currently, there are many research studies on soluble BAFF, but the understanding of membrane-bound BAFF is still not deep enough [16,48]. Our results showed that in the disease process of NMOSD, down-regulation of miR-30b-5p led to increased expression of membrane-bound BAFF, which in turn triggered the secretion of soluble BAFF during protein hydrolysis and affected the immune response process.

This study has two limitations. First, MALAT1 mainly increased the expression of membrane-bound BAFF, but whether it increased the expression of soluble BAFF remains unknown. The molecular mechanism behind it deserves further exploration. Second, more cytological and zoological experimental evidence is needed to confirm whether the downstream effect on the activation and proliferation of B cells in the relapse stage of NMOSD disease is related to the upregulated membrane-bound BAFF content of monocytes.

## 5 Conclusion

In brief, this study suggested that overexpression of MALAT1 could bind and absorb miR-30b-5p in NMOSD monocytes, and then miR-30b-5p reduced the inhibition of BAFF transcription translation, resulting in overexpression of the corresponding receptors of BAFF binding B cells, which may trigger a series of activated B cell proliferation responses. These findings elucidated a novel molecular mechanism by which MALAT1 mediated indirect B cell activation. In addition, it provided an explanation for the over-activation of NMOSD B cells caused by miR-30b-5p down-regulation.

## Supplementary Material

Supplementary material
