# Supplementary material for: Excessive MALAT1 promotes the immunologic process of neuromyelitis optica spectrum disorder by upregulating BAFF expression
Source: Transl Neurosci. 2023 Oct 17;14(1):20220306. doi: 10.1515/tnsci-2022-0306 (PMC10590614; doi:10.1515/tnsci-2022-0306)
Supplement: Supplementary material [file tnsci-2022-0306-sm.pdf]

Supplementary material

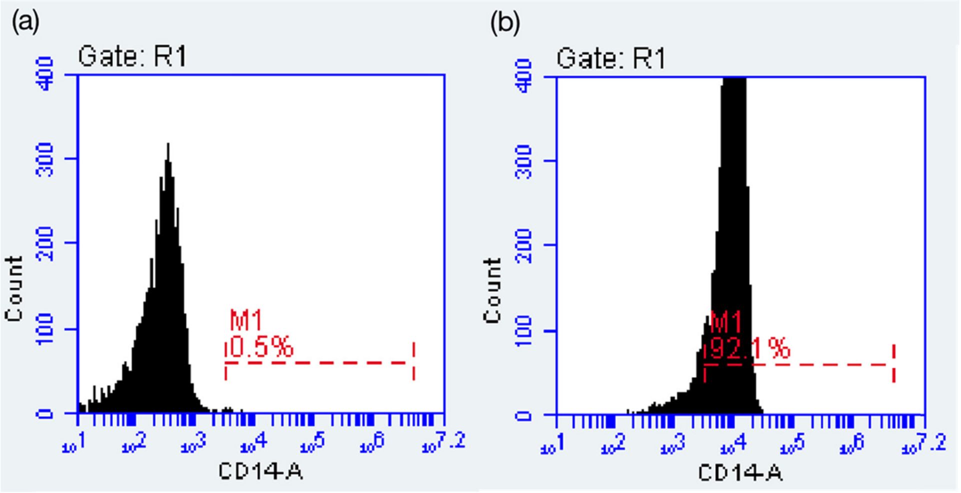

Figure S1: CD14+ cells isolation. (a) Flow analysis before sorting showed very little CD14+ cells. (b) The purity of $CD14^+$ cells after sorting was up to 92.1%.

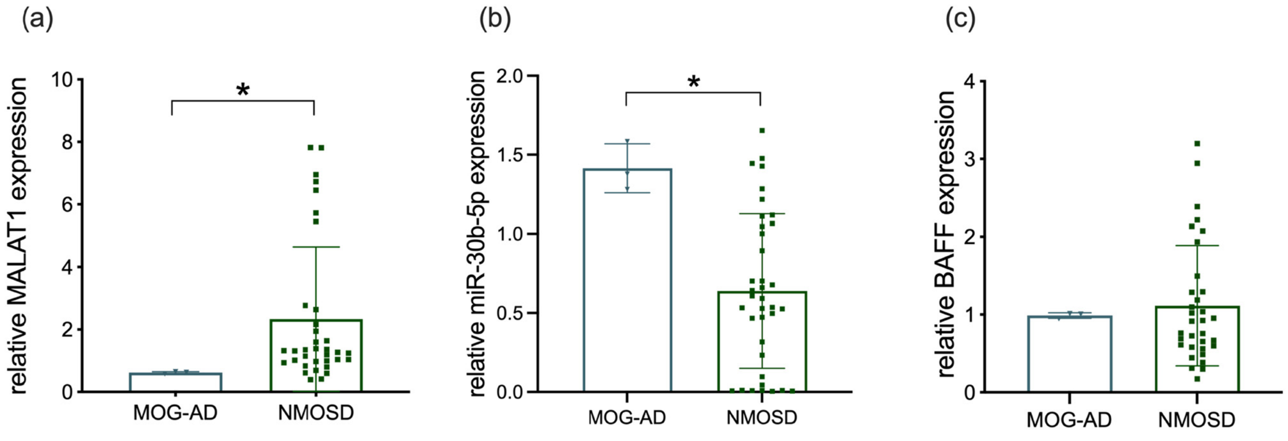

Figure S2: Expression of MALAT1, miR-30b-5p and BAFF in CD14+ monocytes of MOG-AD patients in qPCR assay, comparing with NMOSD patients. (a–c) $2^{-\triangle\triangle CT}$ values of MALAT1, miR-30b-5p and BAFF in CD14+ monocytes in MOG-AD group and NMOSD group, respectively (*$P < 0.05$, **$P < 0.01$, ****$P < 0.0001$).

Table S1: The sequences of miR-30b-5p and miR-30b-5p-mut for Dual-Luciferase assay

NR_002819 (miR-30b-5p)	AGCCCATCAATTTAATTTCTGGTGGTGCAGAAGTTAGAAGGTAAAGCTTGAGAAGATGAGGG**TGTTTAC**<br>GTAGACCAGAACCAATTTAGAAGAATACTTGAAGCTAGAAGGGGAAGTTGGTTAAAAATCACATCAAAAAGC<br>TACTAAAAGGACTGGTGTTTCTTTTTCCCTTAGGTCTGTCTAGAATCCTAAAGGCAAATGACTCAAGGTGTAAC<br>AGAAAACAAGAAAATCCAATATCAGGATAATCAGACCACCACAG**GTTTAC**<br>AGTTTATAGAAACTAGAGCAGTTCTCACGTTGAGGTCTGTGGAAGAGATGTCCATTGGAGAAATGGCTGGTA<br>GTTACTCT
NR_002819 (miR-30b-5p)-mut	AGCCCATCAATTTAATTTCTGGTGGTGCAGAAGTTAGAAGGTAAAGCTTGAGAAGATGAGGG**GTGGGCA**<br>GTAGACCAGAACCAATTTAGAAGAATACTTGAAGCTAGAAGGGGAAGTTGGTTAAAAATCACATCAAAAAGC<br>TACTAAAAGGACTGGTGTTTCTTTTTCCCTTAGGTCTGTCTAGAATCCTAAAGGCAAATGACTCAAGGTGTAAC<br>AGAAAACAAGAAAATCCAATATCAGGATAATCAGACCACCACAG**TGGGCA**<br>AGTTTATAGAAACTAGAGCAGTTCTCACGTTGAGGTCTGTGGAAGAGATGTCCATTGGAGAAATGGCTGGTA<br>GTTACTCT

Table S2: Primer sequences for real-time PCR

	**Forward primer**	**Reverse primer**
MALAT1	AAAGCAAGGTCTCCCCACAAG	GGTCTGTGCTAGTCAAAAGGCA
miR-30b-5p	GCGCGTGTAAACATCCTACAC	AGTGCAGGGTCCGAGGTATT
	GTCGTATCCAGTGCAGGGTCCGAGGTATTCGCACTGGATACGACAGCTGA	
BAFF	TGAAACACCAACTATACAAAAAG	TCAATTCATCCCCAAAGACAT
β-actin	CTGGGACGACATGGAGAAAA	AAGGAAGGCTGGAAGAGTGC
U6	AGAGAAGATTAGCATGGCCCCTG	CAGTGCAGGGTCCGAGGT
	GTCGTATCCAGTGCAGGGTCCGAGGTATTCGCACTGGATACGACAAAATA	
